# Complete Genome Sequence of the Alfalfa Symbiont *Sinorhizobium/Ensifer meliloti* Strain GR4

**DOI:** 10.1128/genomeA.00174-12

**Published:** 2013-02-14

**Authors:** Francisco Martínez-Abarca, Laura Martínez-Rodríguez, José Antonio López-Contreras, José Ignacio Jiménez-Zurdo, Nicolás Toro

**Affiliations:** Grupo de Ecología Genética de la Rizosfera, Estación Experimental del Zaidín, Consejo Superior de Investigaciones Científicas (CSIC), Granada, Spain

## Abstract

We present the complete nucleotide sequence of the multipartite genome of *Sinorhizobium/Ensifer meliloti* GR4, a predominant rhizobial strain in an agricultural field site. The genome (total size, 7.14 Mb) consists of five replicons: one chromosome, two expected symbiotic megaplasmids (pRmeGR4c and pRmeGR4d), and two accessory plasmids (pRmeGR4a and pRmeGR4b).

## GENOME ANNOUNCEMENT

The primary source of biologically fixed nitrogen in crops is found in the symbiotic interaction between legume plants and certain soil microorganisms, collectively referred to as rhizobia (1). *Sinorhizobium* bacteria are the microsymbionts of *Medicago* (e.g., *Medicago sativa* and *Medicago truncatula*), *Melilotus*, and *Trigonella* legume species.

The genome sequences of four *Sinorhizobium meliloti* strains are publicly available (2–4). *S. meliloti* GR4 was isolated as the predominant rhizobial strain (i.e., nearly 50% of the isolates) from an agricultural field with a well-documented crop history in Granada, Spain (5, 6). Besides the chromosome and the expected symbiotic megaplasmids (pRmeGR4c and pRmeGR4d), this strain harbors two accessory plasmids, designated pRmeGR4a and pRmeGR4b. The latter contains a region that was identified as the genetic determinant of the particularly high competitiveness for nodulation on alfalfa roots along with a plethora of mobile genetic elements (7–10).

A highly pure genomic DNA sample of *S. meliloti* GR4 was sequenced on a GS FLX Titanium platform (Roche Diagnostics) at Macrogen, Inc. (South Korea), on the basis of both shotgun and 3-kb paired-end libraries, resulting in 70-fold genome coverage. Raw sequence data fit the quality standards of the Genomes OnLine Database (GOLD) (11). Sequencing reads were *de novo* assembled (Newbler 3.0), resulting in a total of 12 scaffolds (>40 kb each) and 51 contigs (<3 kb each). Most of the gaps (78%) were closed using customized informatics scripts (L. Martínez-Rodríguez, J. A. López-Contreras, F. Martínez-Abarca, and N. Toro, unpublished data). The remaining gaps (except two, corresponding to repeated sequences) were manually closed by combining Southern blot hybridization data and a detailed observation of relevant sequencing reads with the Tablet tool (http://bioinf.scri.ac.uk/tablet/). The genome was annotated using the Integrated Microbial Genomes (IMG) Expert Review (ER) service (12). Replicon sizes and the G+C content of the chromosome and plasmids pRmeGR4a, pRmeGR4b, pRmeGR4c (related to pSymA), and pRmeGR4d (related to pSymB) are 3,618,794 bp (62.8%), 175,986 bp (60.0%), 225,725 bp (58.6%), 1,417,856 bp (60.4%), and 1,701,197 bp (62.4%), respectively. The complete genome consists of 6,700 protein-coding sequences: 3,334 on the chromosome, 1,541 on pRmeGR4d, 1,393 on pRmeGR4c, 247 on pRmeGR4b, and 185 on pRmeGR4a. Similarly to other *S. meliloti* genomes, 3 *rrn* chromosomal operons and 55 tRNA loci (52 on the chromosome and 3 on plasmids) were identified. In addition, 1,066 noncoding RNA genes (sRNAs) were predicted in this genome based on those identified in the *S. meliloti* 1021 and 2011 reference strains (12, 13). Genome comparisons using the MUMmer package (14) revealed a high degree of synteny of the chromosome and the largest plasmid (pRmeGR4d) to the corresponding replicons of the other four sequenced *S. meliloti* strains. This synteny is less pronounced in the symbiotic megaplasmid pRmeGR4c. The two smaller plasmids, pRmeGR4a and pRmeGR4b, did not evidence signs of synteny with any rhizobial genomic region, which, together with their low G+C content, suggests that horizontal transfer has been the major contribution to the mosaic arrangement of these accessory replicons.

### Nucleotide sequence accession numbers.

The accession no. for GR4 chromosome, pRmeGR4a, pRmeGR4b, pRmeGR4c, and pRmeGR4d are CP003933, CP003934, CP003935, CP003936, and CP003937, respectively.
